# The Effect of HRE-Regulated VEGF Expression and Transfection on Neural Stem Cells in Rats

**DOI:** 10.3389/fcell.2020.580824

**Published:** 2020-12-17

**Authors:** Bo Dou, Xiangrong Zheng, Danfeng Tan, Xixi Yin

**Affiliations:** Department of Pediatrics, Xiangya Hospital, Central South University, Changsha, China

**Keywords:** VEGF, transfection, stem cells, hypoxic-ischemic brain damage, hypoxia-responsive element

## Abstract

In this study, we analyzed neural stem cells transfected with the HRE-VEGF gene in groups experiencing different periods of hypoxia. The results of RT-PCR showed that the expression of vascular endothelial growth factor (VEGF) mRNA gradually increased with the prolonged period of hypoxia (*p* < 0.05). The results from the western-blot test showed that expression of the VEGF protein increased with as the period of hypoxia increased (*p* < 0.05). The results of MTT combined with Elisa reagent showed that with the prolonged period of hypoxia, the secretion of VEGF protein increased, and that the proliferation of target cells and neural stem cells was better promoted (*p* < 0.05). These results imply that HRE can safely and effectively regulate VEGF expression. By controlling the period of hypoxia, we can increase the expression level, and limit it in more safe values to avoid the possibility of cancer caused by the over-enhancement of proliferation of target cells due to the overexpression of the VEGF protein.

## Introduction

Hypoxic-ischemic brain damage (HIBD) is a common central nervous system lesion in perinatal neonates, and 25% of neonatal HIBD can develop into severe and permanent neuropsychological sequelae, including developmental delay, cerebral palsy and epilepsy, and there is still no effective therapy for the disease (Liang et al., 2010; Sobrino et al., 2020; Truttmann et al., 2020). Gene therapy for brain damage has become a popular research field, due to its potential for long-lasting and stable effects. However, whether the target gene can have an efficient and regulable expression in a safe context remains an open question in gene therapy. Therefore, it is urgent to find a relatively safe and efficient gene therapy to prevent HIBD.

Vascular endothelial growth factor (VEGF) is a specific mitogen of endothelial cells. It can be secreted to the space outside cells, act on vascular endothelial cells to stimulate the proliferation and migration of cells, and accelerate angiogenesis. It has a direct, protective effect on nerve tissues before the formation of new blood vessels, and can, to some extent, reduce neuronal damage. Additionally, VEGF can promote the proliferation of NSCs cultured outside the body (Zhang et al., 2000; Zheng et al., 2012). HRE is a hypoxic-sensitive enhancer which can significantly enhance the expression levels of downstream genes under hypoxic conditions. There is a *cis*-acting element that can regulate VEGF gene transcription during hypoxia, in the 5′ native promoter region of the VEGF gene. We therefore constructed a VEGF gene fragment, with the HRE promoter in the upstream gene sequence, and transfected this fragment into the NSCs to explore whether HRE can significantly enhance the expression of VEGF under hypoxic conditions.

This experiment aims to construct a recombinant, lentivirus carrying VEGF gene, and a VEGF-gene recombinant lentivirus carrying the HRE, and to transfect two groups of target genes into neural stem cells by lentivirus, to obtain genetically engineered NSCs with expressed VEGF, and culture them in different conditions. Our study revealed that transfection of HRE-VEGF can effectively promote the proliferation of neural stem cells, providing a safe and efficient gene therapy for hypoxic-ischemic brain damage.

## Materials and Methods

### Cell Culture

Take a pregnant Sprague Dawley (SD) rat at 14 gestational days. Use pre-cooling sterile 1 × PBS with a Pasteur pipette to rinse out the meninges and vascular tissues. Add 3 ml DMEM/F12 (1:1) (Gibco, United States) containing 10% fetal bovine serum to a 12-well plate with cell slide. Cells were incubated at 37°C under a 5% CO_2_ atmosphere for 5 days. Renew half of the medium every 2∼3 days according to the growth of the neural stem cells. Place the culture flask under a microscope to observe cell proliferation and growth from multiple fields of vision. After about 7 days of culture, well-formed neurospheres can be observed with a microscope. Continue the subculture according to the steps described above.

### Induced Differentiation of NSCs Outside the Body

On the 5th day of the induced differentiation of NSCs, remove cell slides from the 12-well plate. Cells were blocked with 10% goat serum containing 0.3% Triton X-100 for 2 h and then incubated with primary antibodies overnight at 4°C, then add 0.5 μg/ml DAPI onto the slide to stain the nucleus; gently wash it on a shaker with 1 × PBS. The following primary antibodies were used: 1:100 diluted rabbit anti-GFAP antibody or as the primary antibody and the 1:50 goat anti-rabbit IgG-FITC as the secondary antibody. Images were obtained with Leica fluorescence microscope.

### Construction of pGC-FU-VEGF165 and HRE-pGC-FU-VEGF165 Recombinant Lentiviruses

The corresponding primers were designed according to the CDS region (base sequence: No. 72–650, with 579 bases in total) and the hVEGF165 gene sequence (AF486837) of the HRE gene (No. NM007942.2) released by GENBANK. The primers were synthesized by GENECHEM (Shanghai, China). The primer sequences are displayed as follows. HRE: (Forward) 5′-CGT CTAGAGTCGACGCGGCCGCATCCGCGCCAC-3′ (Reverse) 5′-CGGGAGACTCGAGGCCGGCGTACGACGAC-3′; VEGF: (Forward) 5′-GAGGATCCCCGGGTACCGGTCGCCACCATG AACTTTCTGCTGTCTTGG-3′ (Reverse) 5′-TCACCATGGTG GCGACCGGCCGCCTCGGCTTGTCAC-3′.

### Infecting the Target Cells With Lentivirus

Collect the NSCs in rats under primary culture; add a good amount of complete medium; then repeatedly blow and beat the cell mass with a pipette to disperse the mass as much as possible. Seed about 2 ml of neural stem cells into a 50 ml culture flask at a density of 5 × 10^4^ cells/ml. To determine a better transfection condition with virus, NSCs were transfected with the virus under different multiplicity of infection (MOI) ratios of 0, 1, 50, and 100, while each MOI value condition was controlled with triplicate sets. Expression of GFP was observed under a fluorescence inverted microscope 48 h after transfection. The proportion of transfected cells was detected by flow cytometry and the best MOI value was detected at 50. As shown in Table 1, culture flasks were divided into seven groups with a final concentration of 5 μg/ml: VEGF (NSCs + pGC-FU-VEGF165 Lentivirus), HRE-VEGF (NSCs + 9HRE-pGC-FU-VEGF165 Lentivirus), HRE-VEGF-H6h (NSCs + 9HRE-pGC-FU-VEGF165 Lentivirus + hypoxia 6h), HRE-VEGF-H12h (NSCs + 9HRE-pGC-FU-VEGF165 Lentivirus + hypoxia 12h), HRE-VEGF-H24h (NSCs + 9HRE-pGC-FU-VEGF165 Lentivirus + hypoxia 24h), LV (No-load lentivirus), Ctrl (Control-only NSCs, no lentivirus), (Normoxia: 95%O_2_ + 5% CO_2_; hypoxia: 2%O_2_ + 98%N_2_).

**TABLE 1:** Transfection conditions and grouping.

Group	*NSCs number*	*MOI*	*pGC-FU-VEGF165* Lentivirus *(1* × *10^8^ TU/ml)*	*9HRE-pGC-FU-VEGF165* Lentivirus *(1* × *10^8^ TU/ml)*	No-load lentivirus	Culture conditions
VEGF	10^5^	50	50 μL	0	0	Normal
HRE-VEGF	10^5^	50	0	50 μL	0	Normal
HRE-VEGF-H6h	10^5^	50	0	50 μL	0	Hypoxia 6h
HRE-VEGF-H12h	10^5^	50	0	50 μL	0	Hypoxia 12h
HRE-VEGF-H24h	10^5^	50	0	50 μL	0	Hypoxia 24h
LV	10^5^	50	0	0	50 μL	Normal
Ctrl	10^5^	0	0	0	0	Normal

### Efficiency of Transfection Under Flow Cytometry

Collect cells in a 1.5 ml sterile centrifuge tube and centrifuge at 1000 rpm for 5 min; discard the supernatant liquid. Then add 1–2 ml pasteurized sterile PBS solution to re-suspend the cells; blow and beat and shock the small cell mass to obtain a single-cell suspension; place it on a flow cytometer to detect the rate of GFP-positive cells. Non-transfected NSC groups serve as control groups.

### Expression of VEGF165 Protein in the Neural Stem Cells of Transgenic Rats, Under Western-Blot

According to the sample size, set the ratio of Reagent A to Reagent B at 50:1 and then prepare a proper amount of BCA working solution; add 200 μL BCA working solution to each well and incubate at 37°C for 30 min. Draw a standard curve on the microplate reader; figure out the protein concentration in the standard curve, based on the OD value and dilution ratio of the tested sample. Corresponding to the amount of loading buffer, put the mix (loading buffer with the protein) in boiling water for 10 min to denature; then store it at −20°C for later use. Add 50 μg of the sample to the loading well of the SDS-PAGE gel and observe. After the bromophenol blue enters the separated gel surface, adjust the voltage to 120V; at room temperature, soak the gel plate in a staining tank containing 0.01% Coomassie brilliant blue, and a blue protein band can be observed. Rinse the filter membrane in the TBST solution for 5 min, and then put the filter membrane in 5% skimmed milk; block at room temperature for 3 h; then place the filter membrane in a hybridization bag and add the 1:100 (dilute the antibody with the primary antibody dilution) diluted primary antibody solution; incubate overnight at 4°C. On the following day, wash the filter membrane for 10 min × 3 times with TBST and then put the filter membrane in a new hybridization bag; add the 1:2000 horseradish peroxidase-labeled secondary antibody solution (dilute the antibody with the secondary antibody dilution) and place on a shaker at room temperature for 1 h; wash the membrane for 10 min × 3 times with TBST; remove the water on the membrane with filter paper, and uniformly drip the luminescent liquid on the entire membrane (ECL luminescent liquid, A:B = 1:1); place the filter membrane on the ECL machine for chemiluminescence; take photos and record.

### Expression of Secretory VEGF Protein in Genetically Engineered Neural Stem Cells

Set 7 standard wells; add the standard sample prepared according to the concentration gradient to each well (0.1 ml/well) of a 96-well plate pre-enveloped with VEGF antibody. The well containing only the NSC supernatant liquid is the “zero well.” Add 100 μL of the NSC supernatant liquid to each well using a Finnpipette. Then add 0.01M PBS to each well with the Finnpipette and soak for about 1 min. Add the prepared ABC working solution to each well (0.1 ml/well) with the Finnpipette. Add 0.01M PBS to each well with the Finnpipette; soak for about 2 min and wash five times. Add the TMB coloring liquid which has been maintained at 37°C for 30 min, to each well (90 μL/well) with a Finnpipette and leave to react for 20–25 min at 37°C in the dark. Finally, add the TMB stop liquid to each well (0.1L/well) with the Finnpipette; at this point, the liquid in the well changes from blue to yellow. Read the OD value detected by the microplate reader at 450 nm. Set the zero well to be the control. Subtract the control values of the zero well from all the OD values and then calculate. Establish a standard curve with the OD values of the prepared concentration gradient standard samples (1000, 500, 250, 125, 62.5, 31.2, and 15.6 pg/ml). According to the established standard curve and OD values obtained from the samples, the corresponding VEGF protein concentrations have been confirmed.

### Proliferation of Genetically Engineered Neural Stem Cells Using MTT

Collect neural stem cells and adjust the cell suspension to a suitable concentration. Add the cell solution to each well (100 μL/well) of a 96-well plate. After plating, make the final density of the tested cells to be 5000 cells/well (fill edge wells with sterile PBS). Set 3 repeated wells per sample. Place the cells in an incubator for continuing culture until all wells are covered with cells in a single layer. Observe under an inverted microscope. Add the MTT staining liquid to each well (10 μL/well) and continue to culture for 4 h. Add the Formazan solution to each well (100 μL/well), and then place on a shaker for low-speed oscillation 10–30 min, to ensure the crystal substance is completely dissolved. If some of the Formazan is still not dissolved, you can gently and repeatedly blow and beat 2–3 times with a pipette to dissolve it, but bubbles should be avoided. Measure the absorbance with ELISA at 570 nm (Operate according to the requirements on the MTT cell proliferation and cytotoxicity test kits).

### Statistical Methods

Data were expressed by $\bar{\text{x}}$ ± s. One-way ANOVA and LSD tests were used to determine the significance of difference among groups. A *p*-value less than 0.05 was considered statistically significant. SPSS 16.0 software was used for the statistical analysis.

## Results

### Identification of NSCs by Immunofluorescence

**FIGURE 1:**
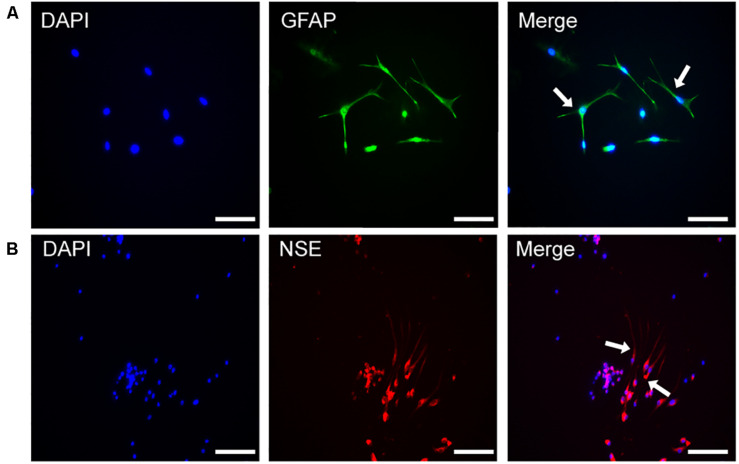
The identification of NSCs by immunofluorescence. **(A)** Immunofluorescence of DAPI and GFAP in the differentiated NSCs. **(B)** Immunofluorescence of DAPI and NSE in the differentiated NSCs, Scale bar: 25 μm.

The nerve cells were placed under a fluorescence microscope and a green fluorescence was observed, which indicated that the neuroglial cell-specific marker GFAP staining was positive (Figure 1A). At the same time, red fluorescence representing the neuron-specific marker NSE, was positive in the differentiated cells (Figure 1B).

### Recombinant Lentivirus, Neural Stem Cell Identification and the Transfection Rate

**FIGURE 2:**
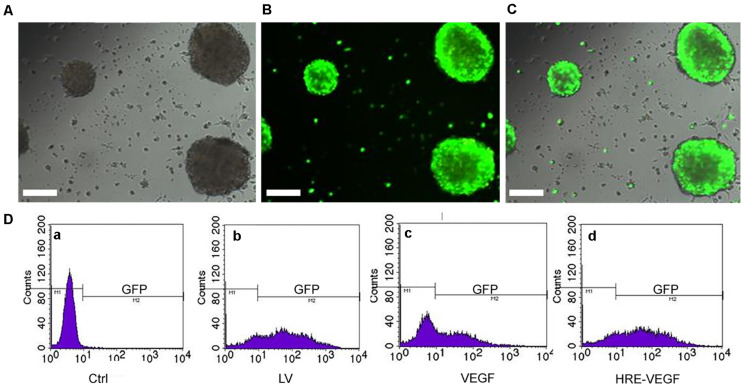
NSCs were transfected by lentiviral. **(A–C)** After transfection with the virus MOI for 50 by 24 h, the green fluorescence could be seen clearly under the fluorescence microscope. Scale bar: 200 μm. **(D)** The efficiency of transfection under flow cytometry: **(a)** Ctrl in normoxia: 0.49%. **(b)** LV: 80%. **(c)** VEGF transfected in normoxia: 50%. **(d)** HRE-VEGF transfected in hypoxia by 6, 12, and 24 h: 80%.

PCR identification was performed on the transfected HRE-pGC-FU-VEGF165 and pGC-FU-VEGF165. Completely consistent with the sequence in the GenBank, HRE-pGC-FU-VEGF165 and pGC-FU-VEGF165 recombinant lentiviruses were successfully constructed. Fluorescence microscopy showed green fluorescence in the genetically engineered neural stem cells in the LV and HRE-VEGF groups, while no green fluorescence was observed in the Ctrl (Figures 2A–C). The results of flow cytometry showed that the transfection rate of cells in group VEGF was about 50%, and the transfection rates in the HRE-VEGF and LV groups were about 80%. The transfection rate in the Ctrl was about 0.49% (Figure 2D).

### The Expression of VEGF165 in NSCs Under RT-PCR

**FIGURE 3:**
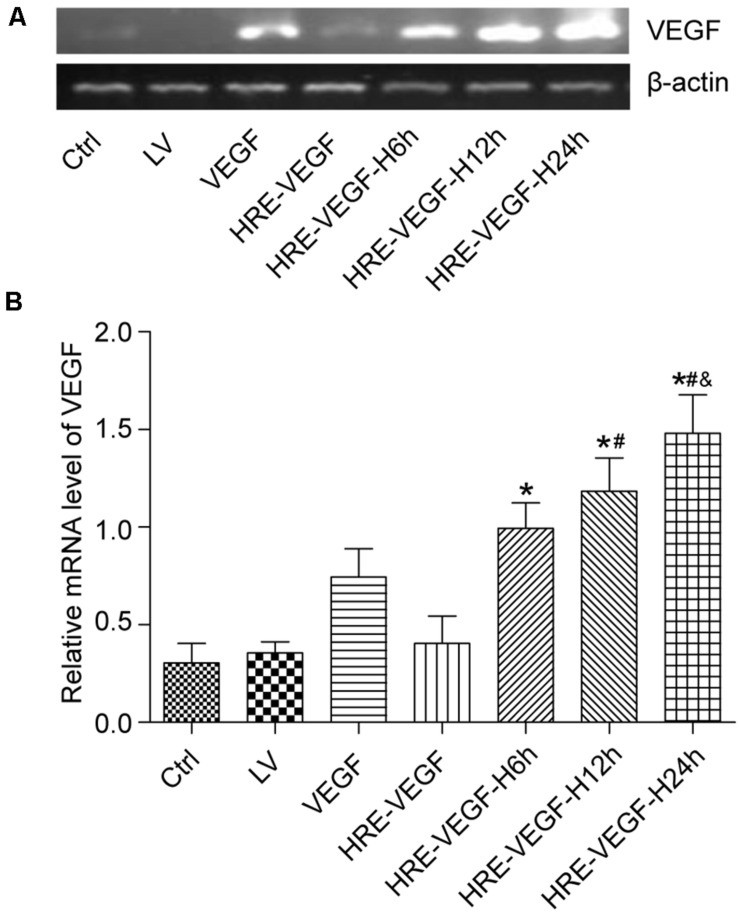
The measured expression of VEGF165 mRNA in each group. **(A,B)** Determination of VEGF165 mRNA by Nucleic acid gel electrophoresis for different transfection conditions in NSCs. Data were expressed as mean ± SD, one-way analysis of variance (ANOVA). (*p < 0.05 compared with HRE-VEGF, ^#^*p* < 0.05 compared with HRE-VEGF-H6h, and ^&^*p* < 0.05 compared with HRE-VEGF-H12h).

Groups HRE-VEGF, LV, and Ctrl exhibited no obvious hybridization bands. The ratios of the HRE-VEGF-H6h, HRE-VEGF-H12h, and HRE-VEGF-H24h groups in pixel element were higher than group VEGF (*p* < 0.05), and the ratio of groups HRE-VEGF-H6h, HRE-VEGF-H12h, and HRE-VEGF-H24h gradually increased with the prolonged period of hypoxia (*p* < 0.05), suggesting that the VEGF165-transfected NSCs could specifically express VEGF mRNA. Under hypoxic conditions, the HRE promoters could significantly increase the expression of VEGF (*p* < 0.05). Also, with the prolonged period of hypoxia, the ratio in pixel element also increased, suggesting that the promoting capacity of the hypoxic HRE promoters gradually increased with the prolonged period of hypoxia (Figure 3).

### The Expression of VEGF165 Protein in NSCs Under Western-Blot

**FIGURE 4:**
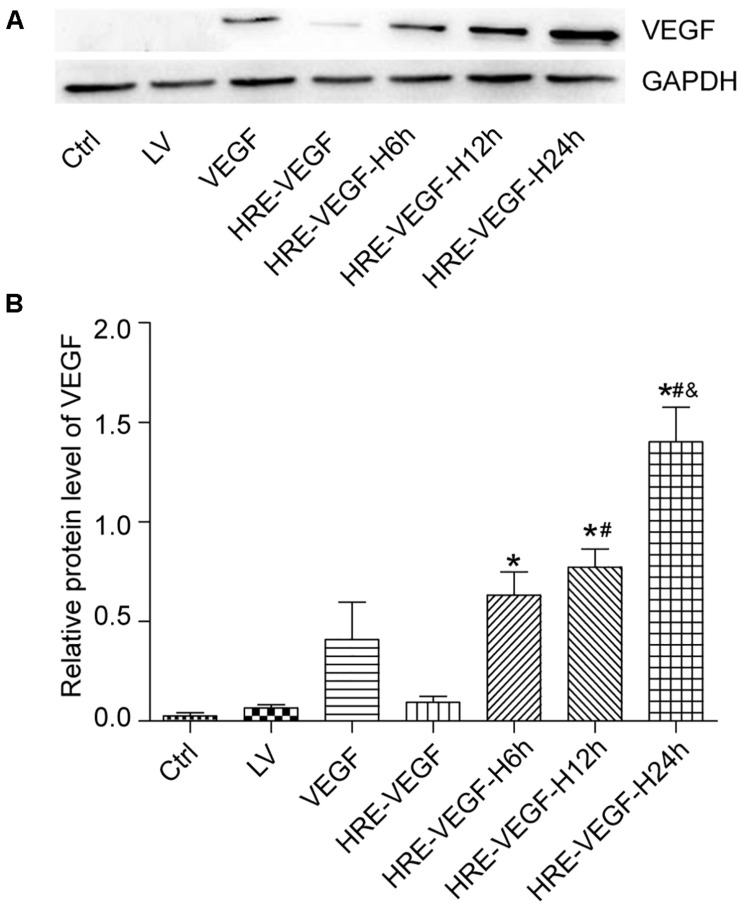
The expression of VEGF165 target gene under western-blot. **(A,B)** Protein expression of VEGF165 by western-blot in NSCs. Data were expressed as mean ± SD, one-way analysis of variance (ANOVA). (*p < 0.05 compared with HRE-VEGF, ^#^*p* < 0.05 compared with HRE-VEGF-H6h, and ^&^*p* < 0.05 compared with HRE-VEGF-H12h).

The ratios of VEGF and internal reference protein bands in gray scale were 1.335 ± 0.016, 1.253 ± 0.025, 1.151 ± 0.027 and 0.950 ± 0.005, respectively. The ratios of groups HRE-VEGF-H6h, HRE-VEGF-H12h, and HRE-VEGF-H24h in gray level were higher than group A (*p* < 0.05), and groups HRE-VEGF-H6h, HRE-VEGF-H12h, and HRE-VEGF-H24h showed increased expression of VEGF protein with the prolonged period of hypoxia (*p* < 0.05). Groups HRE-VEGF-H6h, LV and Ctrl showed no obvious hybridization bands, indicating that the NSCs transfected with the pGC-FU-VEGF165 lentivirus could specifically express VEGF protein. The NSCs transfected with the pGC-FU-HRE-VEGF165 lentivirus could also specifically express VEGF protein under hypoxic conditions, and the expression level was higher than the NSCs transfected with the pGC-FU-VEGF165 lentivirus. The ability of NSCs transfected with the pGC-FU-HRE-VEGF165 lentivirus to express VEGF protein increased with the prolonged period of hypoxia, indicating that the promoting ability of hypoxic HRE promoters gradually increased with the prolonged length of hypoxia (Figure 4).

### Expression of Secretory VEGF Protein in Genetically Engineered Neural Stem Cells Under ELISA

The protein levels in groups HRE-VEGF-H6h, HRE-VEGF-H12h, HRE-VEGF-H24h, and VEGF were significantly higher than in groups HRE-VEGF, LV and Ctrl (*p* < 0.05); the protein level in groups HRE-VEGF-H6h, HRE-VEGF-H12h, and HRE-VEGF-H24h was higher than group VEGF. Also, HRE-VEGF-H6h, HRE-VEGF-H12h, and HRE-VEGF-H24h showed increased protein levels with the prolonged period of hypoxia (*p* < 0.05). There was no significant difference in protein levels among groups HRE-VEGF, LV and Ctrl (*p* > 0.05). These results suggest that the NSCs transfected with the pGC-FU-VEGF165 lentivirus could specifically promote secretion of VEGF protein. The NSCs transfected with the pGC-FU-HRE-VEGF165 lentivirus could also specifically express secretory VEGF protein under hypoxic conditions. In addition, the expression level of secretory VEGF protein of NSCs transfected with the pGC-FU-HRE-VEGF165 lentivirus under hypoxic conditions was higher than the NSCs transfected with the pGC-FU-VEGF165 lentivirus. The ability of NSCs transfected with the pGC-FU-HRE-VEGF165 lentivirus to express secretory VEGF protein, increased with the prolonged period of hypoxia, indicating that the promoting ability of hypoxic HRE promoters gradually increased with the prolonged period of hypoxia (Table 2).

**TABLE 2:** Elisa results of nerve cell supernatant in each group (*N* = 3).

Group	Concentration (pg/ml)
VEGF	389.5 ± 98.3
HRE-VEGF	276.6 ± 110.5
HRE-VEGF-H6h	557.3 ± 21.4^△^^✩^
HRE-VEGF-H12h	908.2 ± 10.2^△^^✩^^★^
HRE-VEGF-H24h	1235.2 ± 91.4^✩^^△^^▲^^★^
LV	235.1 ± 46.2
Ctrl	273.4 ± 125.7

*^✩^Compared with the C and D groups, *P* < 0.05, ^△^Compared with the A groups, *P* < 0.05, ^★^Compared with the B1 groups, *P* < 0.05, ▲Compared with the B3 groups, *P* < 0.05.*

### The Proliferative Viability of Genetically Engineered Neural Stem Cells Under MTT

**FIGURE 5:**
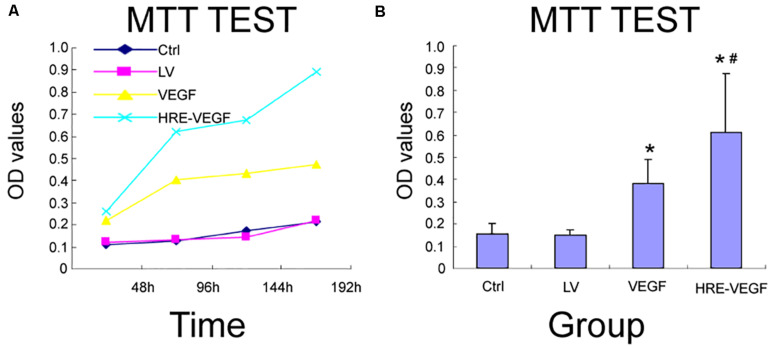
The proliferative viability of genetically engineered NSCs under MTT. **(A,B)** Effects of different transfection conditions on cell viability in NSCs was determined by MMT method. Data were expressed as mean ± SD, one-way analysis of variance (ANOVA). (*p < 0.05 compared with LV, ^#^*p* < 0.05 compared with VEGF).

The OD values for the HRE-VEGF-H6h, VEGF, LV, and Ctrl groups at 490 nm were 0.610 ± 0.261, 0.381 ± 0.110, 0.152 ± 0.436 and 0.155 ± 0.462, respectively. There were no significant differences between the LV and Ctrl groups (*p* > 0.05). There were significant differences among the HRE-VEGF-H6h, LV and Ctrl groups (*p* < 0.05). There were significant differences among the VEGF, LV and Ctrl groups (*p* < 0.05); and there were significant differences between HRE-VEGF-H6h and VEGF (*p* < 0.05). The proliferative viability of cells in groups VEGF and HRE-VEGF was higher than in groups LV and Ctrl (*p* < 0.05). The proliferative viability of cells in the HRE-VEGF group was higher than for the VEGF group (*p* < 0.05). This suggests that transfection of the pGC-FU-VEGF165 lentivirus could promote the proliferation of NSCs. The transfection of the pGC-FU-HRE-VEGF165 lentivirus could also promote the proliferation of NSCs under hypoxic conditions. In addition, the transfection of the pGC-FU-HRE-VEGF165 lentivirus under hypoxic conditions was significantly more effective in promoting the proliferation of NSCs (Figure 5).

## Discussion

Vascular endothelial growth factor, a specific mitogen of endothelial cells, can specifically promote the proliferation, migration and angiogenesis of vascular endothelial cells. It has a direct protective effect on the nervous system before the formation of new blood vessels, and can help prolong the life of cells, reduce neuronal damage, and promote neuronal repair and NSC proliferation (Wei et al., 2017; Li et al., 2019; Sun et al., 2020). We constructed a recombinant lentiviral vector, pGC-FU-VEGF165, carrying VEGF165 gene, and transfected the VEGF gene with the lentivirus into the neural stem cells of fetal rat brain. RT-PCR showed that the NSCs transfected with the VEGF gene could express VEGF mRNA, while the NSCs that were not transfected with the target gene had no significant VEGF mRNA expression. The results of Elisa showed VEGF protein in the medium of neural stem cells transfected with the VEGF gene, and no obvious VEGF protein in the medium of neural stem cells that were not transfected with the target gene, suggesting that the VEGF protein expressed in the neural stem cells transfected with VEGF165 gene could migrate outside the cells. Finally, the results of MTT confirmed that the VEGF protein secreted outside the cells could promote the proliferation of NSCs cultured outside the body, which was consistent with previous studies from our research group.

Efficient gene therapy relies on efficient and stable expression of foreign genes in the receptor. A variety of factors can regulate the expression of the VEGF gene, including hypoxia that has been reported in many studies for the specific upregulation of the VEGF gene (Greenwald et al., 2019; Dzhalilova and Makarova, 2020). Mukhopadhyay et al.’s study has showed that the hypoxia-responsive element (HRE) is an important regulatory sequence mediating the hypoxic responses of cells. The mutation of an HRE site will cause genes in the HRE-regulated downstream sequence of the cell chromosome to lose transcriptional responses to hypoxia (Mukhopadhyay et al., 2000). Therefore, HRE is necessary for hypoxia-induced VEGF expression. We constructed a recombinant VEGF165 gene lentiviral vector 9HRE-pGC-FU-VEGF165 which carries HRE promoters in the upstream region, and transfected the HRE-VEGF gene into neural stem cells in the brains of fetal rats, with lentivirus as the media, to obtain genetically engineered neural stem cells. The results of RT-PCR showed that the genetically engineered stem cells could significantly express VEGF mRNA in a hypoxic environment, and that the expression level was significantly higher than for NSCs transfected with a VEGF gene carrying no HRE promoters (*p* < 0.05). Similarly, the results of the Western-blot test showed that the genetically engineered neural stem cells transfected with the HRE-VEGF gene could significantly express VEGF protein in a hypoxic environment, and that the expression level was significantly higher than for NSCs transfected with a VEGF gene carrying no HRE promoters (*p* < 0.05). The results of the Elisa test showed VEGF protein in the medium of NSCs transfected with the HRE-VEGF gene, and that the protein level was significantly higher than for NSCs transfected with a VEGF gene carrying no HRE promoters (*p* < 0.05), suggesting that, compared with NSCs transfected with a VEGF gene carrying no HRE promoters, NSCs transfected with the HRE-VEGF gene could secrete more VEGF protein outside the cells. The MTT test confirmed that increased secretory protein can significantly enhance the proliferation of NSCs cultured outside the body, consistent with the expected results. But these results still need further study and verify in animal or series other experiments, like apoptosis, function etc.

In addition, under a fluorescence microscope, no obvious green fluorescence was seen in the HRE-VEGF genetically engineered NSCs cultured under normoxia. The results of flow cytometry showed that the transfection rate was about 0.5%. The possible reasons are: (1) The target gene failed to be transfected into the target cell genome. (2) The HRE-VEGF gene was successfully transfected into the target cell genome. However, because cells were cultured under normoxic conditions, the HRE sequence could not be activated, which made the VEGF gene in the downstream region of the HRE and the GFP reporter genes, not able to properly express. Meanwhile, the HRE-VEGF genetically engineered NSCs cultured under normoxia showed no obviously expressed VEGF gene in RT-PCR and Western-blot tests. Whether normoxic conditions prevented the activation of the HRE sequence and thus caused failure in the expression of downstream genes of HRE, still needs further research.

This experiment successfully constructed hypoxic-induced genetically engineered NSCs with highly expressed VEGF genes. Under hypoxic conditions, and affected by HRE promoters, the expression of VEGF factors in the target cells increased significantly and could significantly promote the proliferation of NSCs. In addition, HRE could regulate the expression of VEGF by controlling the period of hypoxia and thus obtain efficient and regulated VEGF transgenic neural stem cells. Our work provides an experimental basis for further *in vivo* experiments on the transplantation of HRE-regulated VEGF-gene-transfected NSCs for HIBD in neonatal rats. Additionally, of particular note is the safety and reliability when we use virus vector, and these questions also should be considered our next study.

## Data Availability Statement

The original contributions presented in the study are included in the article, further inquiries can be directed to the corresponding author.

## Ethics Statement

The animal study was reviewed and approved by the Ethics Committee of Xiangya Hospital.

## Author Contributions

XZ conceived and designed the experiments. DT and BD performed the research, and collected and analyzed the data. DT and XY wrote the manuscript. All authors read and approved the final manuscript.

## Conflict of Interest

The authors declare that the research was conducted in the absence of any commercial or financial relationships that could be construed as a potential conflict of interest.
